# Supplementary figures and images for: Norrisanima miocaena, a new generic name and redescription of a stem balaenopteroid mysticete (Mammalia, Cetacea) from the Miocene of California
Source: PeerJ. 2019 Oct 8;7:e7629. doi: 10.7717/peerj.7629 (PMC6788442; doi:10.7717/peerj.7629)

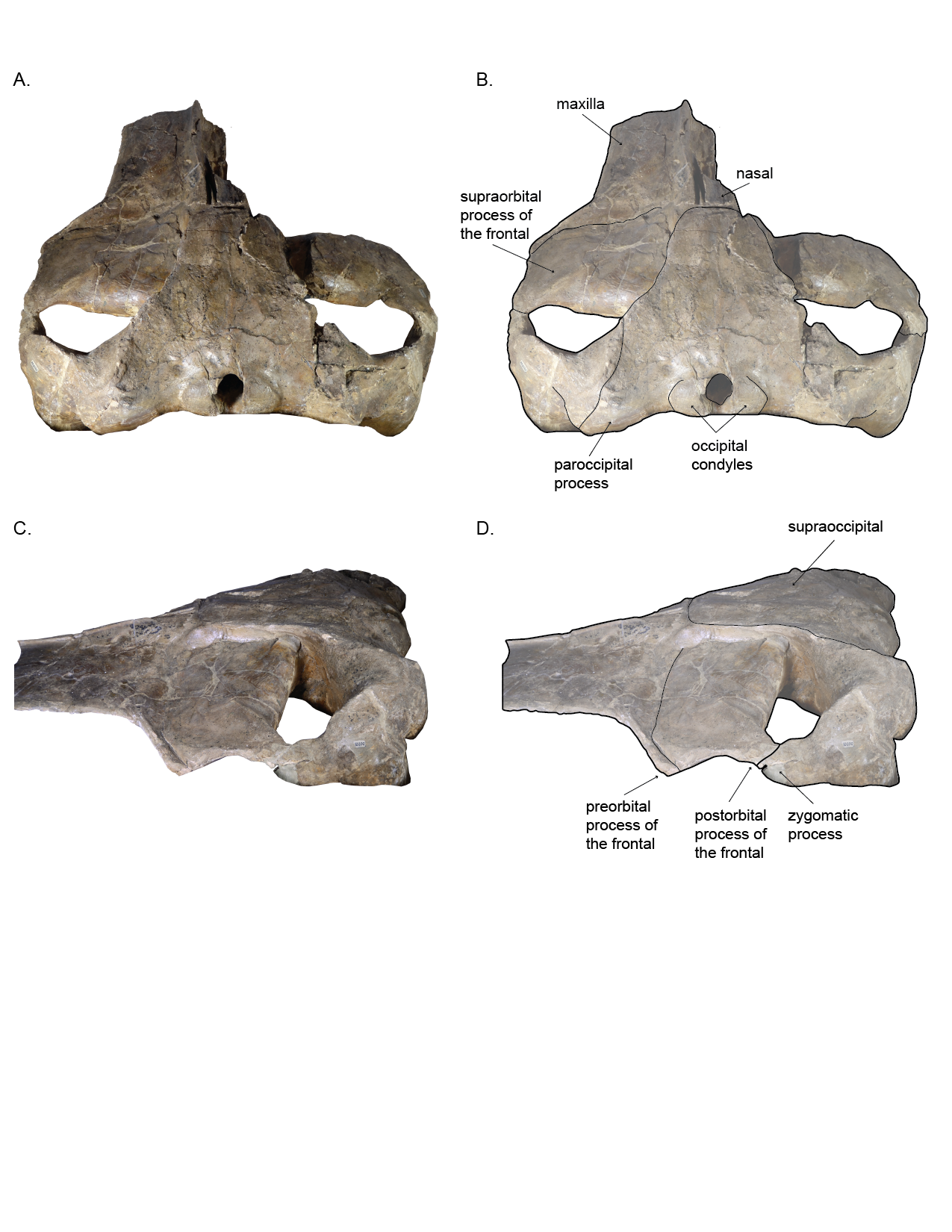

Supplement: Figure S1 — Shown in A,B. Posterior; and C,D. Lateral views. [file peerj-07-7629-s001.png]

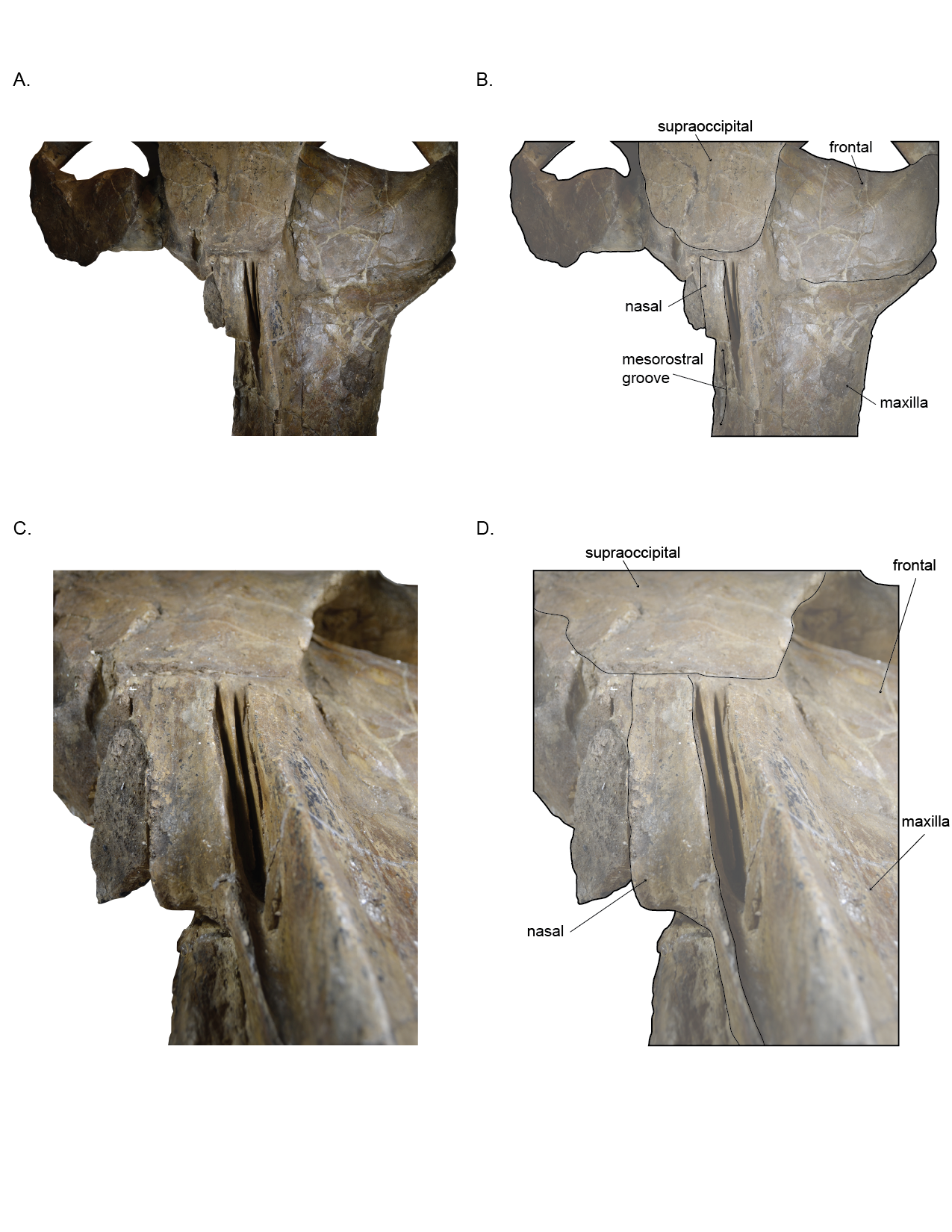

Supplement: Figure S2 — Shown in A,B. Dorsal; and C,D. Anterodorsal views. [file peerj-07-7629-s002.png]

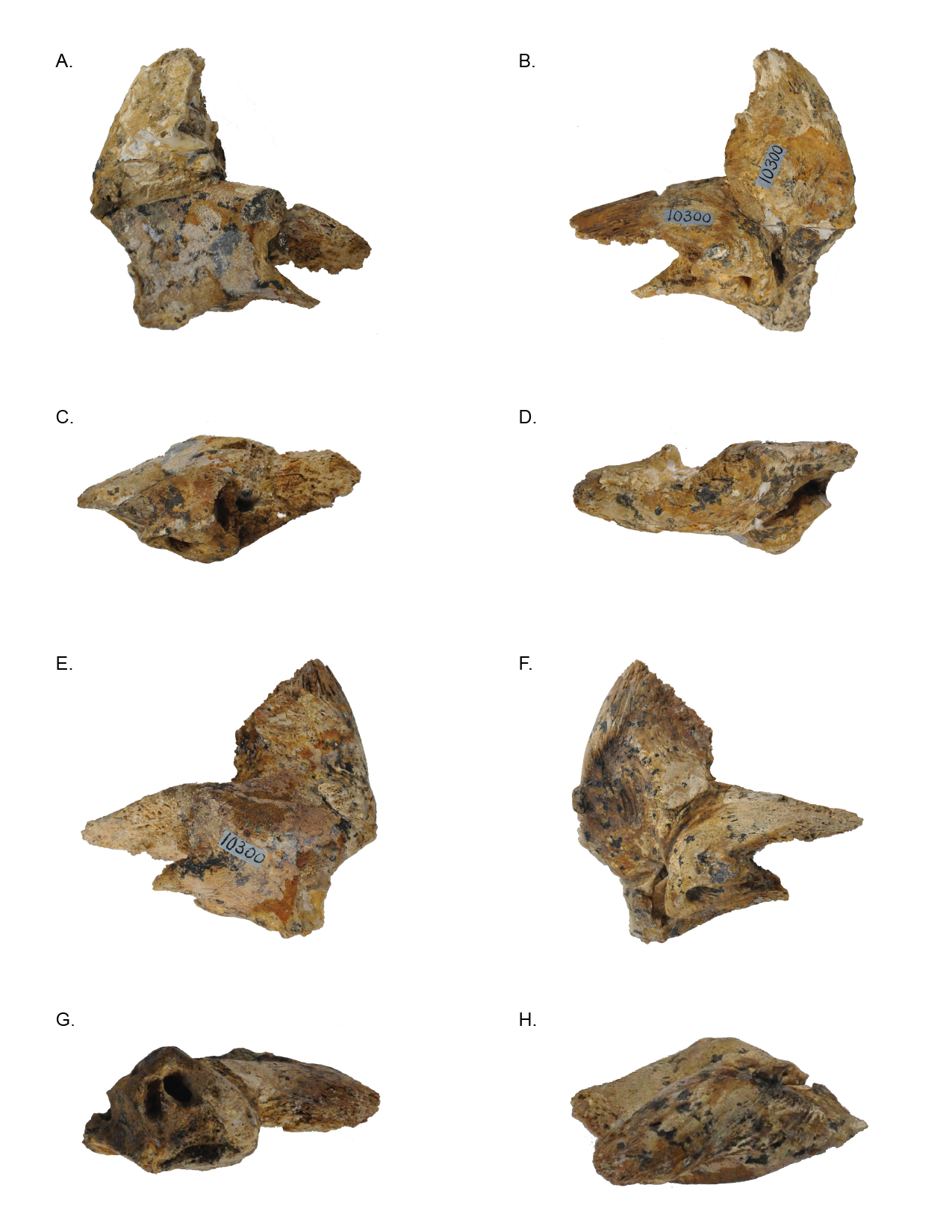

Supplement: Figure S3 — Left periotic shown in A. Dorsal, B. Ventral, C. Medial, and D. Lateral views. Right periotic shown in E. Dorsal, F. Ventral, G. Medial, and H. Lateral views. [file peerj-07-7629-s003.png]

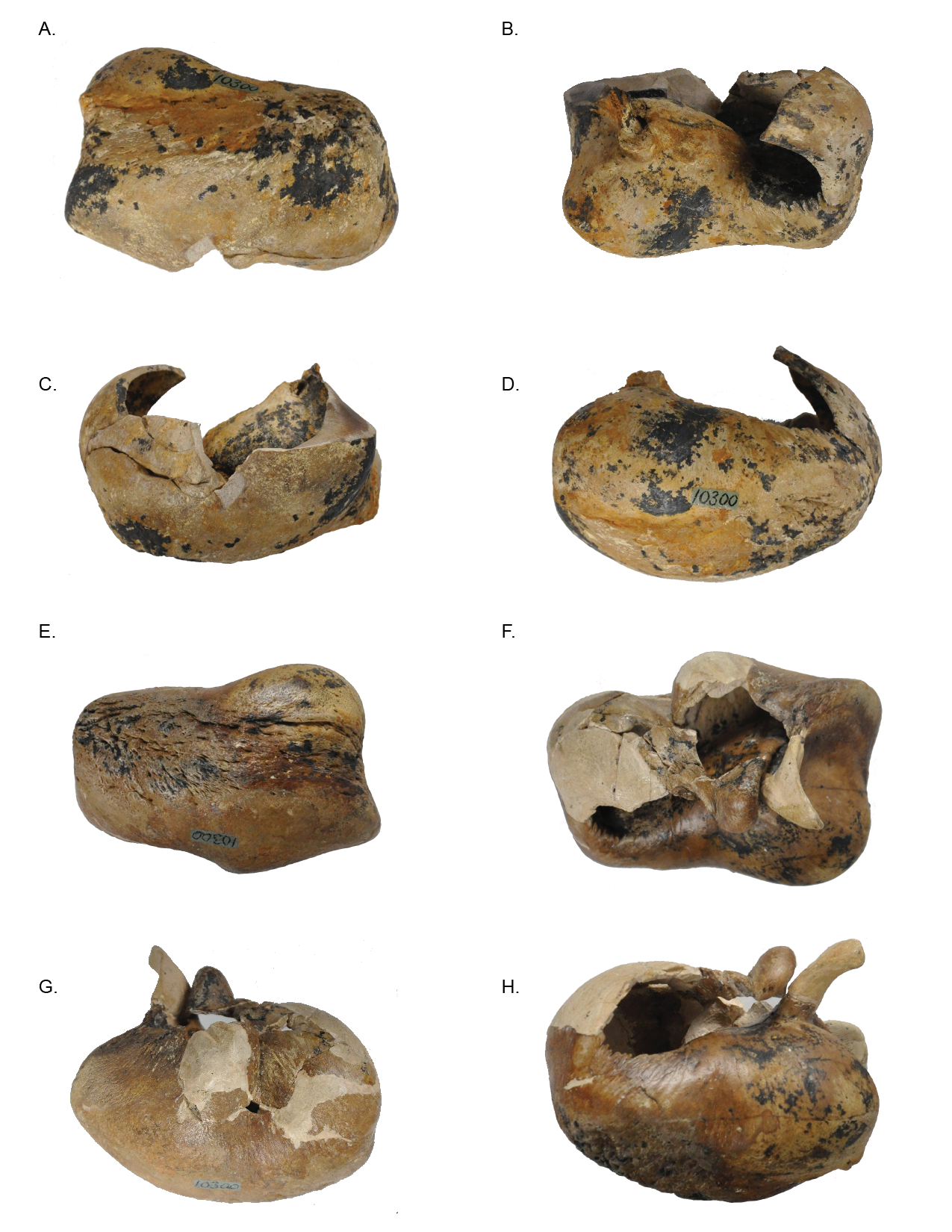

Supplement: Figure S4 — Left periotic shown in A. Ventral, B. Dorsal, C. Lateral, and D. Medial views. Right periotic shown in E. Ventral, F. Dorsal, G. Lateral, and H. Medial views. [file peerj-07-7629-s004.png]

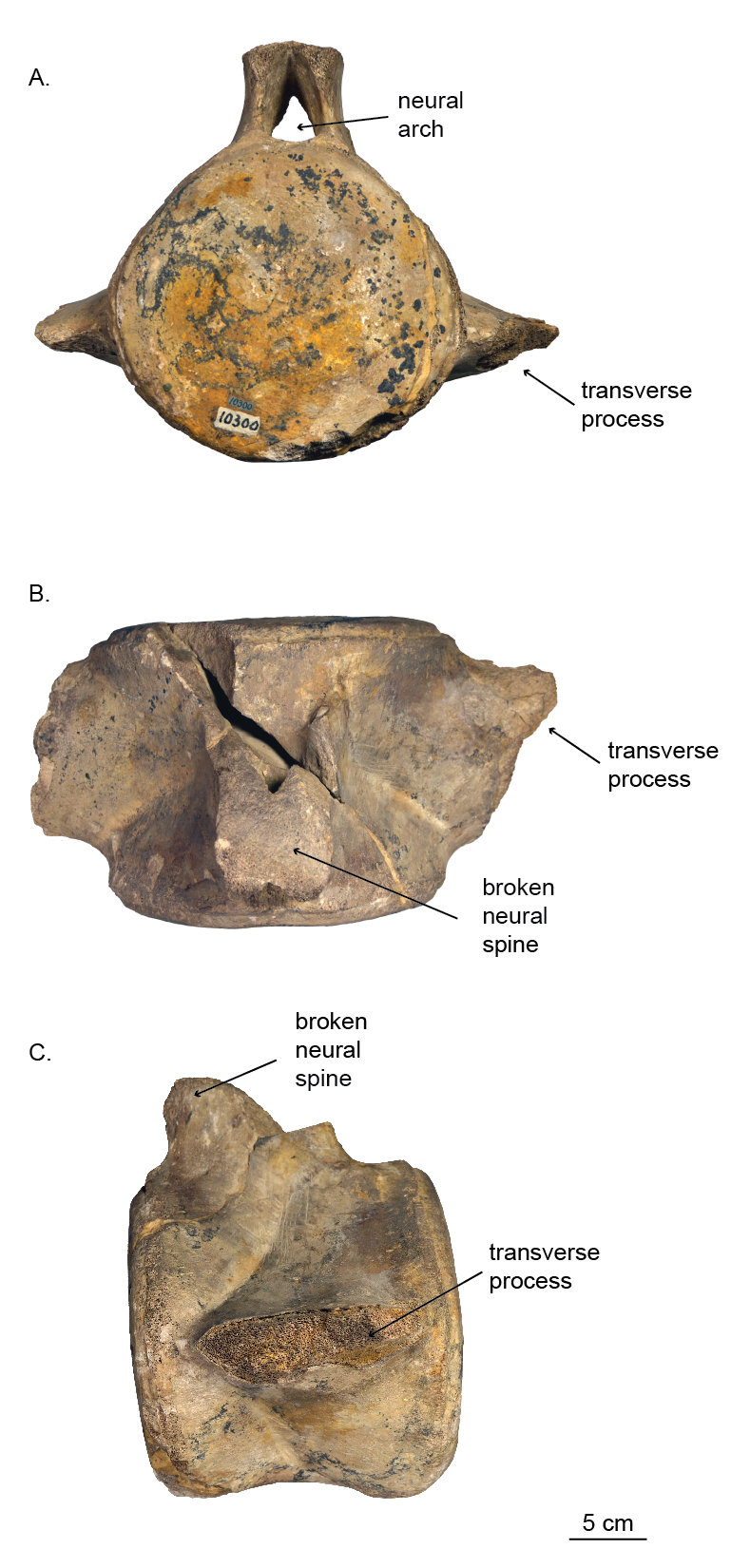

Supplement: Figure S5 — Shown in A. Anterior, B. Dorsal, and C. Lateral views. [file peerj-07-7629-s005.png]

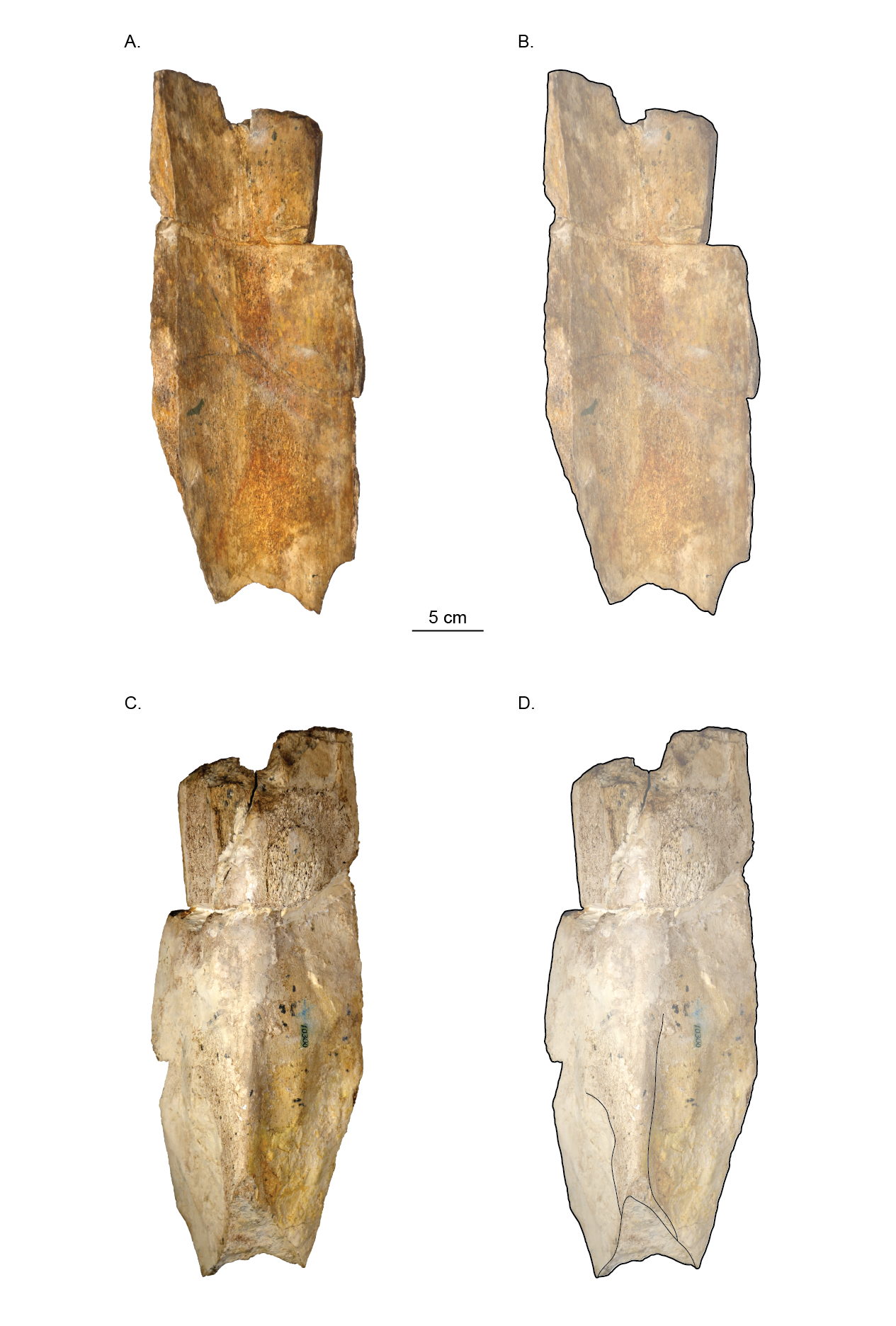

Supplement: Figure S6 [file peerj-07-7629-s006.png]
